# Setting the morphologic quality limits enabling accurate classification of charred archaeological grape seeds

**DOI:** 10.1038/s41598-024-66896-z

**Published:** 2024-07-12

**Authors:** Vlad Landa, Yekaterina Shapira, Adi Eliyahu-Behar, Reut Levitan Ben-Arie, Ehud Weiss, Yuval Reuveni, Elyashiv Drori

**Affiliations:** 1 Department of Computer Science, Ariel University, 40700 Ariel, Israel; 2 Department of Chemical Engineering, Biotechnology and Materials, Ariel University, 40700 Ariel, Israel; 3 Department of the Land of Israel Studies and Archaeology, Ariel University, 40700 Ariel, Israel; 4 Department of Chemical Sciences, Ariel University, 40700 Ariel, Israel; 5 Civil Administration of Judea and Samaria, Ariel, Israel; 6 Archaeobotanical Laboratory and National Natural History Collection of Plants’ Seeds and Fruits, Martin (Szusz) Department of Land of Israel Studies and Archaeology, Institute of Archaeology, Bar-Ilan University, 5290002 Ramat-Gan, Israel; 7 Department of Physics, Faculty of Natural Sciences, Ariel University, Science Park, 40700 Ariel, Israel; 8 Remote Sensing Lab, Eastern R&D Center, 40700 Ariel, Israel; 9 The Samson Family Institute of Grape and Wine Research, Eastern Regional R&D Center, 40700 Ariel, Israel

**Keywords:** Grapevine, Morphometric analysis, Machine learning, Archaeobotanical remains, Computational biology and bioinformatics, Machine learning

## Abstract

This study investigates the morphological changes in grape pips resulting from various charring conditions. Employing high-resolution scanning combined with morphometric measurements for morphological analysis, we aimed to understand the effects of charring on grape pips. Our morphometric analysis demonstrated significant alterations in seed shape above 250 °C. The length–width ratio and the occurrence of cracks notably changed, providing a basis for assessing charring conditions. In addition, applying a machine learning classification method, we determined that accurate classification of grape varieties by the morphometric analysis method is feasible for seeds charred at up to 250 °C and 8 h. Integrating the morphometric changes and temperature ranges suitable for classification, we developed a sorting model for archaeological seeds. By projecting length–width ratios onto a curve calculated from controlled conditions, we estimated charring temperatures. Approximately 50% of archaeological seeds deviated from the model, indicating drastic charring conditions. This sorting model facilitates a stringent selection of seeds fit for classification, enhancing the accuracy of our machine learning-based methodology. In conclusion, combining machine learning with morphometric sorting enables the identification of charred grape seeds suitable for identification by the morphometric method. This comprehensive approach provides a valuable tool for future research for the identification of charred grape seeds found in archaeological contexts, enhancing our understanding of ancient viticulture practices and grape cultivation.

## Introduction

Grapevine is one of the classical fruits of the Old World and an essential part of the oldest group of fruit trees around which horticulture evolved in the Mediterranean basin^1^. This species includes thousands of known cultivars, grown at a wide array of climatic conditions, as well as its wild progenitor (*Vitis vinifera* ssp. sylvestris)^2,3^.

In order to unravel the identity of species and varieties used historically, archaeobotanical remains are collected from excavations and analyzed. Most archaeobotanical assemblages worldwide, including wood and seeds, were preserved due to charring as a result of incomplete combustion in natural or deliberate fires. As the original organic matter chemical structure is dramatically altered by charring—organisms cannot utilize it as an energy resource. Therefore, charred materials are more likely to be preserved in the archaeological record than uncharred organic materials. Thus, charred archaeobotanical remains became an essential component of archaeological excavations, mainly used to reconstruct human subsistence and the climatic conditions at that time. These remains can also be used as a carbon source for radiocarbon dating^2,3^. However, their nucleic acid preservation is badly affected by the charring conditions^4,5^.

Wood and the outer endocarp layer of many seeds comprise cellulose, hemicellulose, and lignin. Cellulose is a polysaccharide chain composed of linked glucose monomers and is considered an almost inexhaustible source of raw material for the increasing demand for environment-friendly and biocompatible products^6^. Lignin is a natural phenolic polymer rich in aromatic groups occurring in higher plant tissues and is the second most abundant polymer after cellulose^7^.

Significant chemical changes take place in plant material during the charring process. As the temperature increases, cellulose, hemicellulose, and lignin, the plant’s major structural components, break down, while water, CO, CO_2_, and other gases are released. As grape seeds comprise 43.8 wt% of lignin, 36.8 wt% of sugar polymers (hemicellulose and cellulose), 17 wt% of extractives, and 2.4 wt% of ash^8–10^, the evaporation of these gases results in condensation and aromatization of the carbon skeleton, which leads to the formation of graphite-like structures^11^ and causes deformations of the physical properties of the seeds^12^. However, little is known about the preservation of charred archaeological plant specimens and the chemical changes and degradation processes they undergo over^13^.

Previously, we have shown that grape seeds can be classified to the varietal level by a morphological classification methodology based on 3D scanning^14^. In addition, we have demonstrated how a machine-learning-based 3D image processing methodology can accurately classify fresh and charred grape seeds to the variety level^15^. The latter constitutes the foundation for the construction of massive charred seed reference libraries, using traditional varieties’ collections, towards a full-scale seed-based variety classification platform for archaeo-botanic grape seed remains. Once established, this method can dramatically change our ability to follow the ancient history of winemaking.

This effort joins the efforts of additional groups, which, in the past few years, sought to develop various machine learning (ML) techniques applied with morphological features to automate the classifications of archeological remains and grapevine seeds^16–18^. More recently, Cervantes et al. proposed the classification of representative Vitis vinifera cultivars, conserved in the Spanish collection of IMIDRA, using methods of image analysis and optimized morphological approaches, based on seeds shape^19^. A similar study presented a classification of grapevine cultivars utilizing the Random Forest (RF) ML method applied with morphological traits^20^. In addition, several efforts have been made in software development, which allows analysis and documentation procedures for archaeological artifacts, such that features of individual artifacts can be recorded, analyzed, and compared within and between contextual assemblages^21^.

The charring duration and temperatures, and the changes archaeological seeds may undergo through postdeposition degradation processes, are influential to the ability to classify charred remains, as charred seeds may become deformed when charred at high temperatures^12^. Thus, it is imperative to set the limits within which a charred specimen will be deemed suitable for classification by the mentioned methods.

The current study aims to develop a new model for selecting archaeological grape seeds suitable for classification by 3D morphometric analysis. To do so, we conducted several controlled charring experiments and studied the following morphometric changes. In addition, the morphological changes were correlated with the breakdown of their chemical structure using FTIR. The collected data is used as an effort to set a model enabling the determination of the charring temperature of grape seeds charred at unknown conditions (archaeological). In parallel, the range of temperatures in which precise classification of varieties is enabled for charred grape seeds, was determined using our previously published ML classification method^15^.

By combining both the charring temperature prediction model, and the range of temperatures enabling classification, we were able to select an appropriate archaeological grape seed specimens suitable for identification by the machine learning method.

## Materials and methods

### Modern plant material

A total of 365 seeds from two cultivars were sampled from the vineyard collection at Ariel University, Israel: the endogenous Israeli variety 292 (Tzuriman S.) from an endogenous collection previously described^22,23^, and Cabernet Sauvignon from the European varieties collection at Ariel University, Israel.

Mature seeds were extracted from ripened grapes from at least three different grapevines. The seeds were washed with water to discard any residual pulp tissue and air-dried for 2 days, then stored in a closed vial until used. Before scanning, each seed was carefully cleaned with brushes and needles from any external tissues coating its crevices, to enable an effective scan of the seed’s morphology.

### Archeological plant material

A total of 100 grape pips from two archeological sites; Shilo (n = 73) and Beit El (n = 27), were used for scanning and measurements. Below is a description of the archaeological context of the specimens used in the study.

*Shilo* is one of the most important Israelite sites in the land of Israel and is considered the site of the Tabernacle. Grape seeds were identified by dry sifting the sediments in three loci. Locus 8561 (Figure S1 – Online Appendix B) is a layer of soil on a floor in a ‘four-room house’ building, dated to the Iron Age IIb (8th C. BCE), based on the associated pottery. The room was paved with small field stones, and many vessels were discovered on its floor, including many bowls, kraters, and cooking pots. It was therefore interpreted to have been used for food preparation and dining. The building was abandoned during the destruction of the Kingdom of Israel^24^. Locus 8593 is a favissa pit (Figure S2 – Online Appendix B), dated to the Iron Age IIa (tenth–ninth centuries BCE). The pit was blocked with two separate layers of fieldstones, and a round stone slab was discovered next to it, likely intended to cover its opening. In it, grapevine and olive seeds were discovered.

Locus 8583 is also a pit. It is bell-shaped in cross-section and hewn into the rock, interpreted as a storage pit. In its bottom, along with various pottery vessels, wheat, olive, and grapevine seeds were discovered.

*Beit El* The site Khirbet Kfar Murr is located in the Beit El mountains, about 30 km north of Jerusalem. Since 2006, ten excavation seasons have been conducted at the site, in which broad settlement activity has been revealed, dating from the eighth century BC to the eighth century AD. Starting as a small farm or a small village during the Iron Age II, it developed into a small town and reached its peak during the Roman period, being one of the many thriving Jewish settlements in northern Judea.

Grape seeds were collected from locus L-6154, a large wine press located at the northeast of the site (Figure S3 – Online Appendix B). The winepress includes a large 7X5 m treading floor, a sedimentation compartment of ca. 1 m^2^, and a collection pool of ca. 2 m^2^, 2.6 m deep, with seven steps descending to its bottom and a pip sediment crevice. A simple calculation by the collection pool’s volume gives the capacity of circa 10 m^3^ of wine in a single batch. Dating by the pottery, the winepress was used until the end of the big Jewish rebellion during the first century AD. The grape pips used in this study were collected by sifting the sediment at the bottom of the collection pool.

All grape seeds were dry sifted from the sediment using a 5 mm sift and kept in dry conditions (20% relative humidity, 20–22 °C) until scanning or other analysis.

### Charring Experiments and Morphometric Analysis

To identify morphological changes in charred specimens and perform machine-learning-based classification of the archaeological pips, we conducted controlled charring experiments using fresh grape pips of Cabernet Sauvignon and Tzuriman S. 292 grape varieties. Following their extraction from the ripe fruit, and cleaning using a teeth brush and a needle, the fresh grape seed specimens were heat-treated in batches of 20 seeds at a time, at temperatures ranging from 200 to 350 °C with increasing periods between 2 and 8 h in a controlled electric furnace (DFO-240, MRC Ltd. Israel). To simulate actual charring conditions, pips were covered with a thick layer of quartz sea sand to keep low oxygen availability and prevent their burning (oxidation)^12,25–27^.

Additional charring experiments were conducted to track and characterize the chemical changes occurring during charring, using FTIR spectroscopy. Three to five seeds of each variety were heat-treated at temperatures ranging from 150 to 300 °C, with increasing time periods between 2 and 24 h, in the same manner described above. Details of the FTIR analysis and the results obtained are summarized in Supplementary file A.S1.

Table 1 summarizes the experiment conditions. Note that at 150 °C, there seemed to be no point in running the experiment for 24 h due to almost no difference in morphological parameters between 1, 4, and 8 h of charring, while at 300 °C, the 8 h of treatment already showed highly deformed pips. Out of each charring term, two seeds were selected for FTIR analysis.

**Table 1:** Summary of charring experiments conditions.

Temperature (°C)	Duration of heating (h)
150	1, 4, 8
200	1, 4, 8, 24
250	1, 4, 8, 24
300	1, 4, 8

### Morphometric analysis

**Figure 1:**
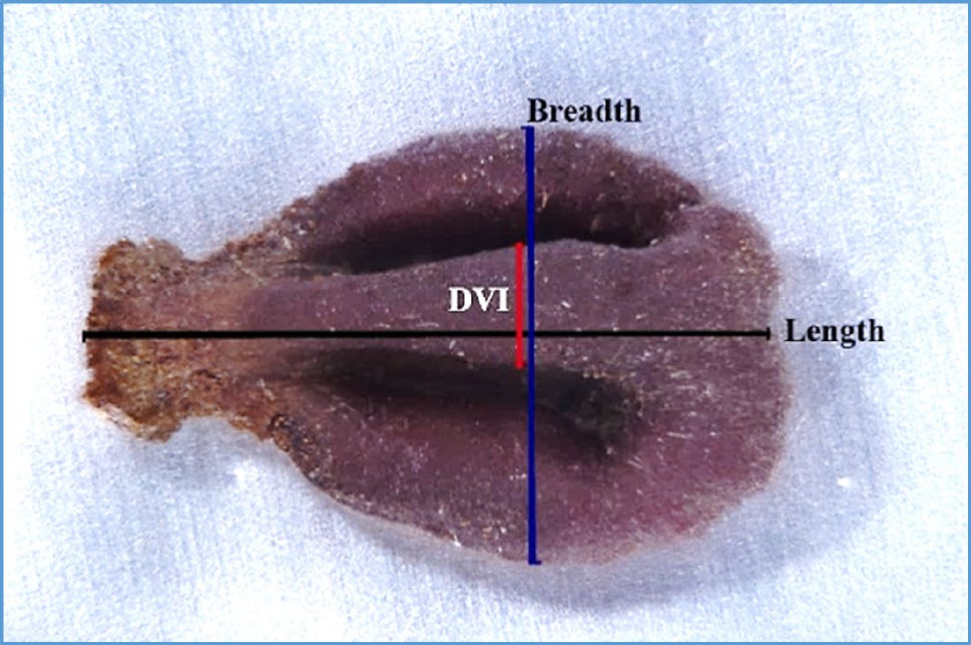
Measurements of length (black line), breadth (blue line) and distance between the ventral infolds (DVI—red line).

In order to quantify the changes in grape seed proportions caused by the charring, we performed a morphometric analysis of each pip before and after charring. We measured the length (black line), the breadth (blue line), and the distance between ventral infolds (red line) on the same line of breadth, as represented in Fig. 1. The selection of the simple morphometric parameters length and width as well as the distance between infolds is due to two considerations, a) preliminary observations we made indicated that the main change following charring of pips is their swelling, dramatically changing these parameters and their ratios. And b) by specifically examining the ratios between width/length and width/DVI (Distance between the Ventral Infolds), we eliminate the differences within a group of seeds from the same variety, caused by different pip sizes. The measurements were conducted using the NIS-Elements D software, whereas the pips were scanned using a Nikon SMZ25 stereomicroscope (Nikon, Tokyo, Japan) equipped with a Nikon DS-Ri2 microscope camera, as described previously^15^. Such quantification constitutes an important step toward the sortation of archeological seeds in terms of suitability for classification under the proposed methodology.

### Linear discriminant analysis (LDA) classification

Following the presented classification methodology in our previous work^15^, we first preprocess every stereoscopic pip scan by transforming it into a cloud points representation. In turn, each pixel and its corresponding height value of the scan were scaled by the intrinsic matrix, which consists of the intrinsic parameters of the scanning device. Once we acquired a cloud point for each scanned pip, we formed a data set of cloud points and split it into training and testing datasets. Then, for each data set, we built a similarity matrix. The training set similarity matrix was constructed by applying an Iterative Closest Point^28^ (ICP) algorithm between every pair of cloud points pips (pip [i] and pip [j]) and populating the matrix with the ICP distance Mean Square Error (MSE) result at index [i,j]. Constructing the testing similarity matrix utilizes the same process, except for the pips cloud points pairs. In this case, the similarity matrix constitutes a combination between the training set and the testing set. Finally, these similarity matrices were used as training and testing sets that were fed into the Linear Discriminant Analysis^29,30^ (LDA) machine learning model. The LDA model learns the inner and outer classes of the ICP similarities in the training pips, and based on the learned features, it is used to classify the testing pips. In order to achieve proper feature extraction, we normalized the training similarity matrix to zero mean and unit standard deviation, and those parameters were used to normalize the testing matrix as well.

In addition, we implemented a tournament methodology described in our previous publication^15^, conducting multiple classification sessions, while at every session we used different tests and training splits to maximize the approximation of the classification accuracy distribution.

Furthermore, an additional normalization factor was used- presenting the scanning pips with equal length while preserving their structural ratio. In such a way, the extracted features were solely based on the morphological differences rather than the physical size.

### Statistical analysis

Morphometric differences following the charring at different times and temperatures were conducted using a Two-way ANOVA test^31^, followed by the Tukey post hoc test^32^, using all 20 seeds per treatment. The statistical analysis was conducted using NIS-Elements D software, JMP Pro 15.1.0 Statistical Software (SAS Institute Inc., Cary, NC, USA: https://www.jmp.com/en_us/home.html) to determine the statistical significance of differences between the treatment means at α = 0.05.

### Plant guideline statement

The experiment with plant and archaeological materials complied with relevant institutional, national, and international guidelines and legislation.

## Results

As elaborated in the introduction, our primary aim is to develop a model enabling the selection of archaeological grape seeds suitable for classification using the 3D ML tool we previously developed. Thus, we first set out to develop a model for estimating seed charring temperatures using a morphometric approach.

### Characterizing the morphological changes in grape seeds upon charring in various conditions

**Figure 2:**
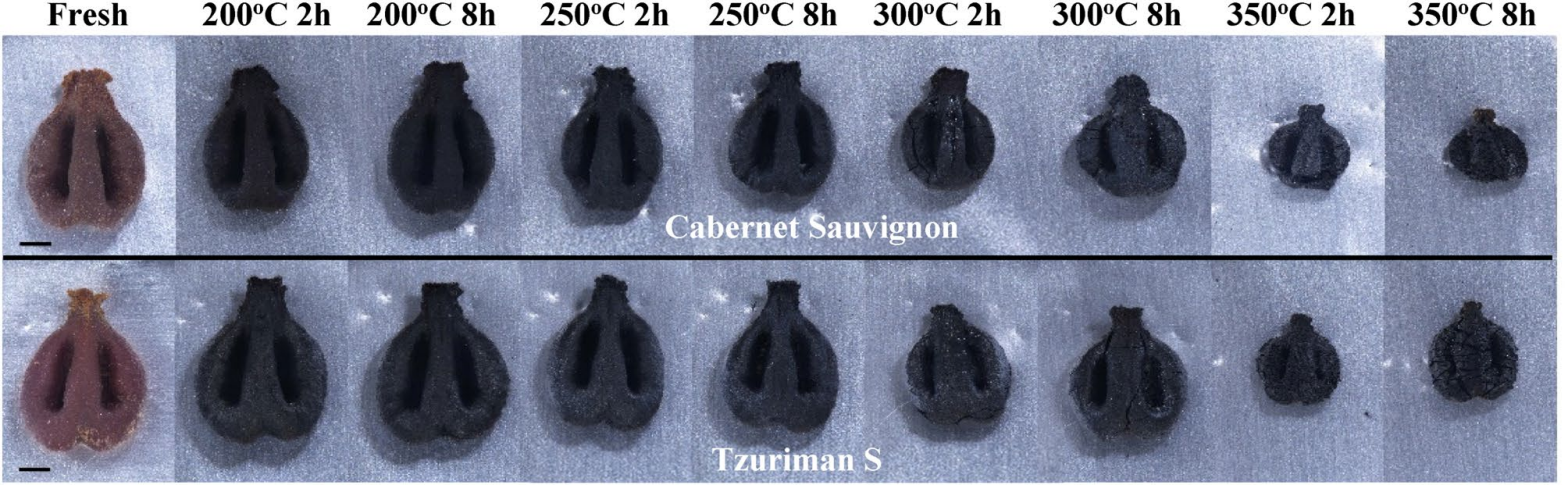
Cabernet Sauvignon (top row) and Tzuriman S. (bottom row) pips before and after charring at different temperatures (from 200 to 350 °C) for different times (2 h and 8 h) (the scale bar is 1 mm).

The charring experiments generally reveal that the pip size is reduced, and its structure is distorted with increasing temperature and duration. Figure 2 presents photos of Tzuriman S. and Cabernet Sauvignon pips before and after charring at increasing temperatures from 200 °C and 2 h to 350 °C and 8 h. The deformations are clearly dependent on the charring temperature (and duration). Note, that pips charred at 200 °C only show a slight reduction in width-to-length ratio with respect to the fresh, uncharred seed, while at temperatures of 250 °C and 300 °C, both cultivars become shorter and rounder, as can be deduced by the decrease in the length/width ratio. After heating for 2 and 8 h at 300 °C, noticeable cracks developed on the pip’s surfaces (Fig. 2). These changes become even more pronounced at a temperature of 350 °C—a significant reduction in the length of the pips is observed, and many cracks developed on their surface. These results are well in agreement with the breaking of chemical bonds and decomposition of the organic matter as was observed by FTIR (See Supplementary file A.S1). In addition, we statistically analyzed the three morphometric parameters, width, breadth, and DVI, for both varieties, utilizing the two-way ANOVA test. Table 2 summarizes the results of the morphometric analysis.

**Table 2:** Morphometric changes occurred during charring of Tzuriman S. and Cabernet Sauvignon seeds.

Charring conditions	Cabernet Sauvignon				Tzuriman S			
	Length (mm)	Width (mm)	DVI	L/W	Length (mm)	Width (mm)	DVI	L/W
Fresh pip	5.81 ± 0.2 A	3.81 ± 0.13 A	0.96 ± 0.1 BC	1.52 ± 0.08	5.83 ± 0.27 A	4.27 ± 0.21 A	1.14 ± 0.09 B	1.36 ± 0.07
200 °C 2 h	5.57 ± 0.21 B	3.65 ± 0.13 AB	0.95 ± 0.09 BC	1.52 ± 0.07	5.61 ± 0.23 AB	4.04 ± 0.19 BC	1.11 ± 0.08 B	1.39 ± 0.07
200 °C 8 h	5.31 ± 0.27 BC	3.54 ± 0.15 B	0.95 ± 0.08 BC	1.49 ± 0.09	5.62 ± 0.38 AB	4.08 ± 0.2 AB	1.21 ± 0.09 B	1.38 ± 0.08
250 °C 2 h	5.17 ± 0.29 CD	3.48 ± 0.2 B	1 ± 0.09 BC	1.48 ± 0.09	5.4 ± 0.27 BC	3.88 ± 0.23 CD	1.15 ± 0.12 B	1.39 ± 0.08
250 °C 8 h	4.97 ± 0.22 D	3.43 ± 0.19 B	0.89 ± 0.07 CD	1.47 ± 0.13	5.28 ± 0.2 C	3.78 ± 0.16 D	1.16 ± 0.08 B	1.40 ± 0.08
300 °C 2 h	4.20 ± 0.24 E	3.18 ± 0.3 C	1.19 ± 0.32 B	1.31 ± 0.10	4.66 ± 0.25 D	3.76 ± 0.21 DE	1.48 ± 0.14 A	1.24 ± 0.09
300 °C 8 h	4.46 ± 0.24 F	3.45 ± 0.28 B	1.4 ± 0.26 A	1.31 ± 0.12	4.45 ± 0.36 D	3.56 ± 0.21 E	1.28 ± 0.21 B	1.29 ± 0.11
350 °C 2 h	3.2 ± 0.19 G	2.7 ± 0.2 D	1.01 ± 0.18 BC	1.19 ± 0.15	3.51 ± 0.22 E	2.9 ± 0.2 F	1.21 ± 0.15 B	1.25 ± 0.11
350 °C 8 h	2.7 ± 0.19 H	2.43 ± 0.24 E	1.12 ± 0.21 BC	1.13 ± 0.14	3.27 ± 0.24 E	2.73 ± 0.24 F	1.14 ± 0.16 B	1.13 ± 0.13

The averages were compared via the Tukey-Kramer post hoctest, where different letters represent results which are statistical different, significance defined as p < 0.05.

### Determining the range of charring conditions enabling precise classification of grape varieties

To assess the impact of the morphological deformations that occurred due to various charring conditions, on the classification accuracy, we conducted a set of training and testing classifications on the two grape varieties, CS and TS, utilizing the machine learning methodology described previously. For each classification, we selected twenty seeds charred at 220 °C for 2 h, from the following varieties: 9043, 98, 138, Cabernet Sauvignon, and Tzuriman S, as a train set. We selected these charring conditions as the minimal temperature which gave full charring on one hand, but where the pips still maintain most of their initial structure, and thus were found to classify with very high accuracy to their correct variety on the other. Moreover, these conditions likely simulate the archaeological conditions of low temperature/low oxygen fires (such as cooking installations) used in other works^26^.

The test sets for each of the classification round consisted of twenty Cabernet and Tzuriman S. seeds charred at the various charring temperatures and times as aforementioned in Table 2.

**Figure 3:**
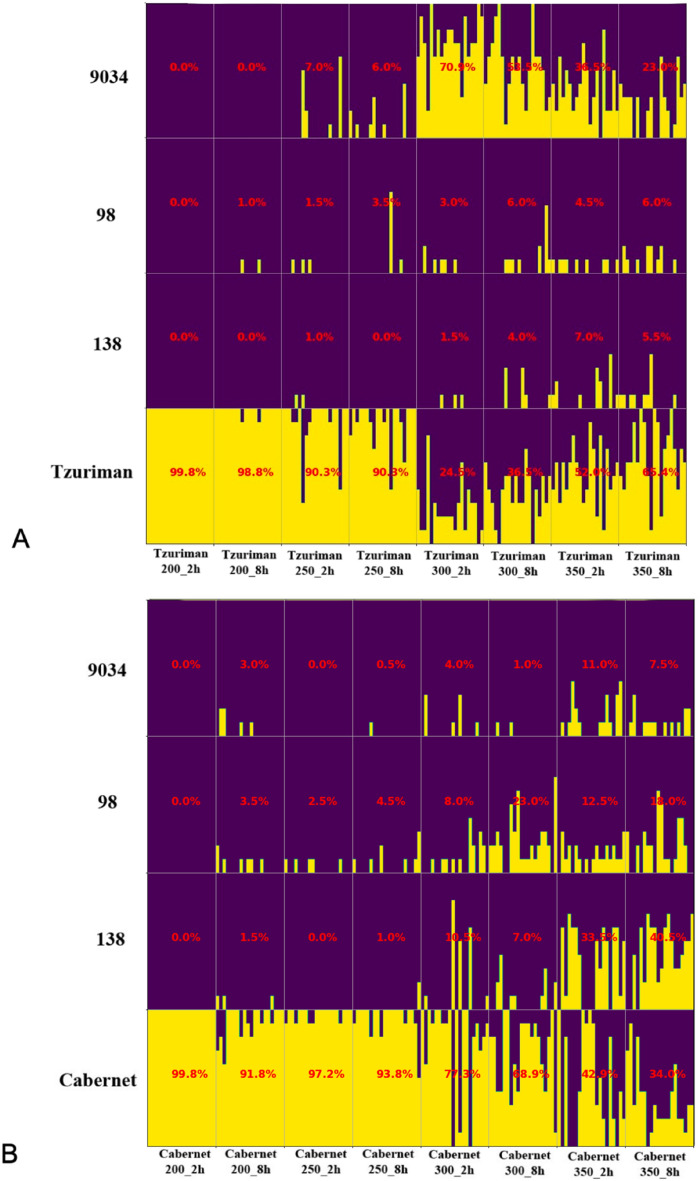
Classification of charred pips, using retraining of the train set pips at every iteration from groups 9043, 98, 138, Cabernet, and Tzuriman S. that was charred at 200 °C for 2 h**.** The y-axis shows the accumulative accuracy of LDA classification after performing 100 training on 20 pips and 100 independent test evaluations of the 20 pips of the groups presented in the x-axis. At every iteration, the twenty pips of Tzuriman S. (**A**) and Cabernet Sauvignon (**B**) that were charred at different temperatures and times were classified independently.

Figure 3A presents the classification statistics for Tzuriman S. pips over 100 separate runs. Classification accuracy of 99.8% and 98.8% were received for pips charred at 200 °C for 2 and 8 h, respectively. The classification results for charred seeds at 250 °C for 2 and 8 h both received accuracies of 90.3%. However, when the charring temperature was raised to 300 °C, the Tzuriman S. classification statistics degraded to 24.5% and 36.5% for 2 and 8 h of charring duration, respectively. Under those conditions, the majority of the pips were misclassified to variety 9034 with 70.9% and 53.5% rates, respectively. At 350 °C, the classification statistics were lower than at 200 °C and 250 °C, but higher than at 300 °C (52.0% and 65.4% for 2 and 8 h of charring, respectively). This alternation of accuracy can be explained by the fact that we introduced the length normalization during the preprocessing stage. Such normalization treats the scans with equal length while keeping their inner ratio. Most likely, the deformation under 350 °C shrunk the seeds enough to project a similar inner ratio to 200 °C, while the 300 °C deformations presented more similarities in ratio with the 9034 variety.

For the Cabernet Sauvignon variety, the results are more intuitive, indicating degrading classification accuracy with the increasing of charring temperature (Fig. 3B). High classification results of 99.8% and 91.8% were received for 200 °C and 250 °C, respectively, independent of charring duration. However, at 300 °C, a decrease in the classification accuracy was observed, reaching 77.3% and 68.9% for 2 and 8 h charring durations, respectively. At 350 °C and 8 h, the lowest classification result of 34.0% was achieved, and most of these pips were misclassified to the 138 variety.

Thus, the results indicate that for both the Cabernet Sauvignon and Tzuriman varieties, the classification accuracy starts degrading when charred at 300 °C for 2 h. Based on these observations, we determine that charring conditions enabling reasonable classification accuracies range from 200 °C and a duration of 2 h up to 250 °C for 8 h.

### Estimating charring conditions of archaeological seeds based on morphometric analysis

Once we established both the morphometrical ratios for charred seeds in each combination of time and temperature, and on the other hand, determined the range of temperatures enabling effective and accurate classification, the two parameters were combined to establish a filtering model for grape seeds with unknown charring conditions, such as archaeological grape seeds, defining subsets of seeds which are suitable for identification using the machine learning classification method.

The statistical examination of a set of 100 archeological grape pips collected from Shilo and Beit-El, reveals that the average length, width, and DVI of these pips are 4.86 ± 0.41 mm, 3.46 ± 0.28 mm, and 1.1 ± 0.21 mm, respectively. This set’s average length/width and width/DVI ratios are 1.4 and 3.14, respectively.

**Figure 4:**
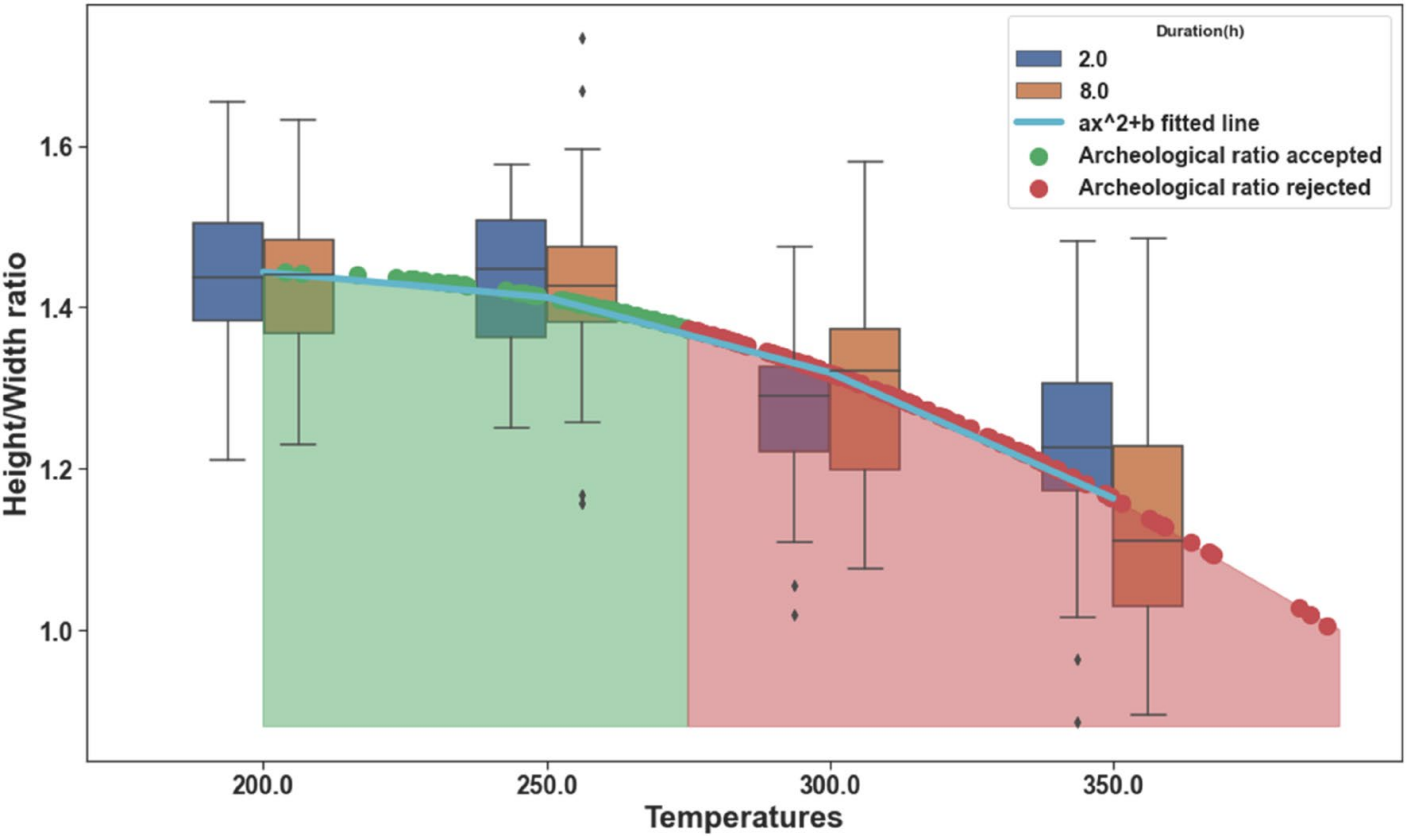
Archaeological seeds’ sorting for classification, using their height/width ratio. Statistic plot of Cabernet Sauvignon and Tzuriman charring morphometric changes based on temperatures (x-axis) and durations of 2 h (blue) and 8 h (brown). The fitted − 0.28x^2^ + 1.44 curve (cyan) represents the morphometric changes in length/width ratio as a function of temperature. Red and green dots represent the projected archeological seeds’ ratios onto the fitted curve, either at accepted temperatures for classification (green dots) or rejected (red dots).

In parallel, we utilized the morphometric results we measured for the fresh seeds charred at the range of temperatures and times to estimate the morphometric changes model. For that purpose, we applied a least square method to fit the *a*x^2^ + *b* function (the model) to the charred seeds’ mean length/width ratio values based on different temperatures and durations. The fitting result estimated parameter “a” to be − 0.28 and parameter “b” to be 1.44 (Fig. 4). Finally, we projected each archeological seed’s length/width ratio onto the fitted curve model to estimate its charring temperature and duration. The resulting projection revealed the estimated charring conditions of the archeological seed, and the temperature (x-axis) is used to indicate whether the selected seed’s ratio holds the conditions to be passed toward ML classification. Using that approach, out of the 100 archeological seeds that were tested, 54 seeds were found to fall in the range which enables further classification (42% out of Beit El site and 58% out of Shilo site), while 46 archaeological seeds were found unsuitable for further classification (58% out of Beit El site and 42% out of Shilo site) (Fig. 4), a few archaeological pips had a very low height/width ratio of about 1, and were probably charred by very high temperatures or altered otherwise, likely by post-deposition degradation (see Figure S3 in supplementary file A).

## Discussion and conclusions

In this study, we aimed to investigate the chemical and morphological alterations grape pips undergo under various charring conditions, to study the classification efficiency at each condition, and to combine both observations to develop a filtering model for archaeological charred grape pips suitable for classification.

The morphometric analysis reveals a relative alteration in shape and morphometric features as a function of charring temperatures and durations. In addition, the results show that a dramatic fall in length–width ratio occurs at charring temperatures above 250 °C, as well as the occurrence of cracks, implicating a dramatic change in seed shape. Thus, the morphometric measurements constitute a reasonable basis for charring conditions assessments for samples charred at unknown conditions, such as archaeological grape seeds.

The second part of the model design involved the determination of the range of temperatures in which precise classification of varieties is enabled for charred grape seeds, using our previously published machine learning classification method^14^. The highest accuracy classification results were achieved for seeds charred at 200 °C and 2-h duration, and up to 250 °C and 8-h duration for both cultivars, which in turn determine the temperatures and durations ranges suitable for reliable classification. These results are in concert with our observation that up to 250 °C, only very slight change to length and breadth of the seeds is observed. But, increasing the temperatures directly influences the level of distortion- the shape of grape seed is changed and they become shorter, rounder and deformed. These temperature-dependent deformations, also reported in other works for grains^25^, as well as for charred grapes seeds^19,20,27^, are probably the cause for the low accuracy achieved when trying to use the 3D structure data for classification by the machine learning method. A reasonable explanation is that the algorithm is unable to match the tested specimen with the train group which maintains the basic characteristics of the fresh pips.

Utilizing both findings; the charring conditions estimation using the morphometric changes, and charring conditions ranges suitable for classification, we established a sorting model for the charred archeological seeds collected at two Biblical-associated archaeological sites- Beit El and Shilo. The sorting was conducted based on their estimated charring temperature, by projecting their length/width ratio onto the curve calculated using the Tzuriman and Cabernet Sauvignon seeds charred by controlled conditions, and by confining the selection to the part of the curve corresponding to up to about 275 °C. An overall of about 50% of the archaeological seeds failed to fit the model, and thus are estimated to have been charred at drastic charring conditions (or by post-deposition degradation), which exclude them from fitting for the machine learning-based classification method. The model enables the selection of higher stringency, thus selecting even a narrower fraction of archaeological seeds fit for classification with higher classification confidence.

Overall, we are confident that combining our previously established machine learning-based classification methodology, using a library of all relevant grape varieties of a specific region, charred by moderate conditions as a train set, and a subset of charred archaeological grape seed, selected by our presented sorting model as fit for classification, will enable accurate identification of charred archaeological seeds to the variety level in future work.

### Supplementary Information

Supplementary Information 1.

Supplementary Information 2.

## Data Availability

The datasets used and analyzed during the current study available from the corresponding author on reasonable request.

## References

[1] Zohary D, Hopf M, Weiss E (2012). Domestication of Plants in the Old World.

[2] Weiner S (2010). Microarchaeology.

[3] Wi SG (2007). Effects of gamma irradiation on morphological changes and biological responses in plants. Micron.

[4] Ramos-Madrigal J (2019). Palaeogenomic insights into the origins of French grapevine diversity. Nat. Plants.

[5] Wales N (2016). The limits and potential of paleogenomic techniques for reconstructing grapevine domestication. J. Archaeol. Sci..

[6] Klemm D, Heublein B, Fink H-P, Bohn A (2005). Cellulose: Fascinating biopolymer and sustainable raw material. Angew. Chem. Int. Edn..

[7] Ciolacu D, Oprea AM, Anghel N, Cazacu G, Cazacu M (2012). New cellulose–lignin hydrogels and their application in controlled release of polyphenols. Mater. Sci. Eng. C.

[8] Moldes D, Gallego PP, Rodríguez Couto S, Sanromán A (2003). Grape seeds: The best lignocellulosic waste to produce laccase by solid state cultures of *Trametes hirsuta*. Biotechnol. Lett..

[9] Brebu M, Yanik JALE, Uysal T, Vasile C (2014). Thermal and catalytic degradation of grape seeds/polyethylene waste mixture. Cell. Chem. Technol..

[10] Yedro FM (2014). Hydrothermal hydrolysis of grape seeds to produce bio-oil. RSC Adv..

[11] Nishimiya K, Hata T, Imamura Y, Ishihara S (1998). Analysis of chemical structure of wood charcoal by X-ray photoelectron spectroscopy. J. Wood Sci..

[12] Charles M, Forster E, Wallace M, Jones G (2015). “Nor ever lightning char thy grain”^1^: Establishing archaeologically relevant charring conditions and their effect on glume wheat grain morphology. STAR Sci. Technol. Archaeol. Res..

[13] Cohen-Ofri I, Weiner L, Boaretto E, Mintz G, Weiner S (2006). Modern and fossil charcoal: Aspects of structure and diagenesis. J. Archaeol. Sci..

[14] Karasik A, Rahimi O, David M, Weiss E, Drori E (2018). Development of a 3D seed morphological tool for grapevine variety identification, and its comparison with SSR analysis. Sci. Rep..

[15] Landa V (2021). Accurate classification of fresh and charred grape seeds to the varietal level, using machine learning based classification method. Sci. Rep..

[16] Szűgyi-Reiczigel Z, Ladányi M, Bisztray GD, Varga Z, Bodor-Pesti P (2022). Morphological traits evaluated with random forest method explains natural classification of grapevine (*Vitis*
*vinifera* L.) cultivars. Plants.

[17] Grosman L (2022). Artifact3-D: New software for accurate, objective and efficient 3D analysis and documentation of archaeological artifacts. PLoS ONE.

[18] Maaten, L., Boon, P., Lange, G., Paijmans, H. & Postma, E. Computer vision and machine learning for archaeology. (2007).

[19] Smith H, Jones G (1990). Experiments on the effects of charring on cultivated grape seeds. J. Archaeol. Sci..

[20] Bouby L (2016). Back from burn out: are experimentally charred grapevine pips too distorted to be characterized using morphometrics?. Archaeol. Anthropol. Sci..

[21] Cervantes E, Martín-Gómez JJ, Espinosa-Roldán FE, Muñoz-Organero G, Tocino Á, Cabello-Saenz de Santamaria F (2021). Seed morphology in key Spanish grapevine cultivars. Agronomy.

[22] Shecori S (2022). A field collection of indigenous grapevines as a valuable repository for applied research. Plants (Basel).

[23] Drori E (2017). Collection and characterization of grapevine genetic resources (*Vitis vinifera*) in the Holy Land, towards the renewal of ancient winemaking practices. Sci. Rep..

[24] Livyatan Ben Arie R (2022). Shiloh in the Iron Age 2 (1000–700 BC): Updated Archaeological Review.

[25] Mangafa M, Kotsakis K (1996). A new method for the identification of wild and cultivated charred grape seeds. J. Archaeol. Sci..

[26] Styring AK (2013). The effect of charring and burial on the biochemical composition of cereal grains: Investigating the integrity of archaeological plant material. J. Archaeol. Sci..

[27] Ucchesu M (2016). Predictive method for correct identification of archaeological charred grape seeds: Support for advances in knowledge of grape domestication process. PLoS ONE.

[28] Besl PJ, McKay ND (1992). A method for registration of 3-D shapes. IEEE Trans. Pattern Anal. Mach. Intell..

[29] Fisher, R. Linear Discriminant Analysis. (1936) 10.4018/9781591408307.ch003.

[30] Mikat, S., Weston, J., Scholkopft, B. & Mullert, K.-R. Fisher discriminant analysis with kernels. In *Neural networks for signal processing IX: Proceedings of the 1999*, ieeexplore.ieee.org. (1999).

[31] Yates F (1934). The analysis of multiple classifications with unequal numbers in the different classes. J. Am. Stat. Assoc..

[32] Tukey JW (1949). Comparing individual means in the analysis of variance. Biometrics.

