# Supplementary material for: Setting the morphologic quality limits enabling accurate classification of charred archaeological grape seeds
Source: Sci Rep. 2024 Jul 12;14:16148. doi: 10.1038/s41598-024-66896-z (PMC11245623; doi:10.1038/s41598-024-66896-z)
Supplement: Supplementary file 1 — Supplementary Information 1. [file 41598_2024_66896_MOESM1_ESM.docx]

**Not all archaeological specimens are equal- Setting the morphologic quality limits enabling accurate classification of charred archaeological grape seeds**

Vlad Landa^1^, Yekaterina Shapira^2^, Adi Eliyahu-Behar^3,4^, Reut Levitan Ben-Arie*, Ehud Weiss^5^, Yuval Reuveni^,6,7*^ and Elyashiv Drori^2,8*^

**Supplementary File A- Fourier Transform Infrared Spectroscopy (FTIR)**

FTIR was used to correlate between the chemical and structural changes that occurred during charring and the morphological observations. Representative spectra were obtained by grinding a few tens of micrograms of charred pips using an agate mortar and pestle. The ground sample was then mixed with IR-Grade KBr, to produce a 5 mm diameter pellet using a press. Spectra were collected between 4000 and 400 cm^−1^ at 4 cm^−1^ resolution for 32 scans using a Thermo iS5 spectrometer. Analysis and interpretations of spectra were conducted at the Laboratory for Archaeological Materials, based on the lab FTIR standards library and by comparison to other published data.

Charring experiments were conducted in the same manner as described in the text; Three to five fresh pips of Cabernet Sauvignon and Tzuriman S. grape varieties were heat-treated at temperatures ranging from 150 to 300°C with increasing periods between 2 to 24 hours in a controlled electric furnace (DFO-240, MRC Ltd. Israel). To simulate actual charring conditions, the pips were covered with a thick layer of quartz sea sand to keep low oxygen availability and prevent their burning (oxidation).

**Figure S1** shows the FTIR spectra of modern Tzuriman S. and Cabernet Sauvignon seeds after heating at 150^o^C for 2 hours under charring conditions (covered with sand), compared to the spectra of a fresh Cabernet Sauvignon seed. At this temperature, no structural changes were observed. The two seed varieties share very similar spectra, corresponding to a mixture of the three primary biomass components: cellulose, hemicellulose, and lignin. These correspond to a mixture of alkene, esters, aromatics, ketone, and alcohol, with different oxygen-containing functional groups observed, e.g., OH (3400–3200 cm^-1^), C=O (1765–1715 cm^-1^), C–O–C (1270 cm^-1^), and C–O–(H) (1050 cm^-1^), etc. In general, the highest IR absorbance of OH and C–O is associated with cellulose; hemicellulose contains higher C=O compounds, whereas lignin is rich in methoxyl– O–CH_3_, C–O–C stretching and C=C stretching of aromatic ring containing compounds that absorb strongly at the fingerprint region of 1800-730 (Yang, et al., 2007; Yedro, et al., 2014; Boukir, et al., 2018).

**Figure S2** presents series of spectra of the two cultivars, Cabernet Sauvignon and Tzuriman S. obtained after charring at increasing temperatures. The gradual structural and chemical changes that the pips undergo during charring, can be followed by their corresponding FTIR spectra. First to be noticed is the decrease in the broad absorption around 3400 cm^-1^, and the disappearance of the small peak at 1010 cm^-1^ attributed to the stretching vibration of bonded hydroxyl groups in crystalline cellulose, hemicelluloses and lignin. At the fingerprint region of 1800–700 cm^-1,^ a similar decrease in peak intensities is generally observed, indicating the breaking of chemical bonds during charring. However, peaks originally associated with lignin seem less affected, such as the 1745 cm^-1^ peak associated with ketone (C=O) groups. These findings indicate the breaking of chemical bonds during the charring of the seeds.

In contrast to the modern charred seeds, the spectra of three archeological seeds show very different characteristics (**Figure S3**). Here, the major absorption peaks are located around 3400, 1570, 1384, and 1037 cm^-1^, and cannot be directly assigned to any of the biomass components, cellulose, hemicellulose, and lignin. Cohen-Ofri et al. (2006) showed that the two prominent peaks at ca. 1570 and 1384 cm^-1^ can be assigned to carboxylate groups formed in fossil charcoal as a result of oxidative diagenesis. A small peak at 1034 cm^-1^ (here appearing at 1037 cm^-1^) was not surely assigned; however, the authors suggested that it might be related to adsorbed clay from the deposition environment. Thus, it is observed that the "original" chemical fingerprint, that might have been indicative of the charring temperature, is "masked" by postdeposition alterations, and does not allow to determine the archaeological specimens charring temperature.

**References**

Boukir, A., L. Hajji, and B. Zghari. "Effect of moist and dry heat weathering conditions on cellulose degradation of historical manuscripts exposed to accelerated ageing: 13C NMR and FTIR spectroscopy as a non-invasive monitoring approach." J. Mater. Environ. Sci 9 (2018): 641-654.

Yang, Haiping, Rong Yan, Hanping Chen, Dong Ho Lee, and Chuguang Zheng. "Characteristics of hemicellulose, cellulose and lignin pyrolysis." Fuel 86, no. 12-13 (2007): 1781-1788.

Yedro, Florencia M., Juan García-Serna, Danilo A. Cantero, Francisco Sobrón, and María J. Cocero. "Hydrothermal hydrolysis of grape seeds to produce bio-oil." Rsc Advances 4, no. 57 (2014): 30332-30339.

Cohen-Ofri, I., Weiner, L., Boaretto, E., Mintz, G. & Weiner, S. Modern and fossil charcoal: aspects of structure and diagenesis. J Archaeol Sci 33, 428–439 (2006).


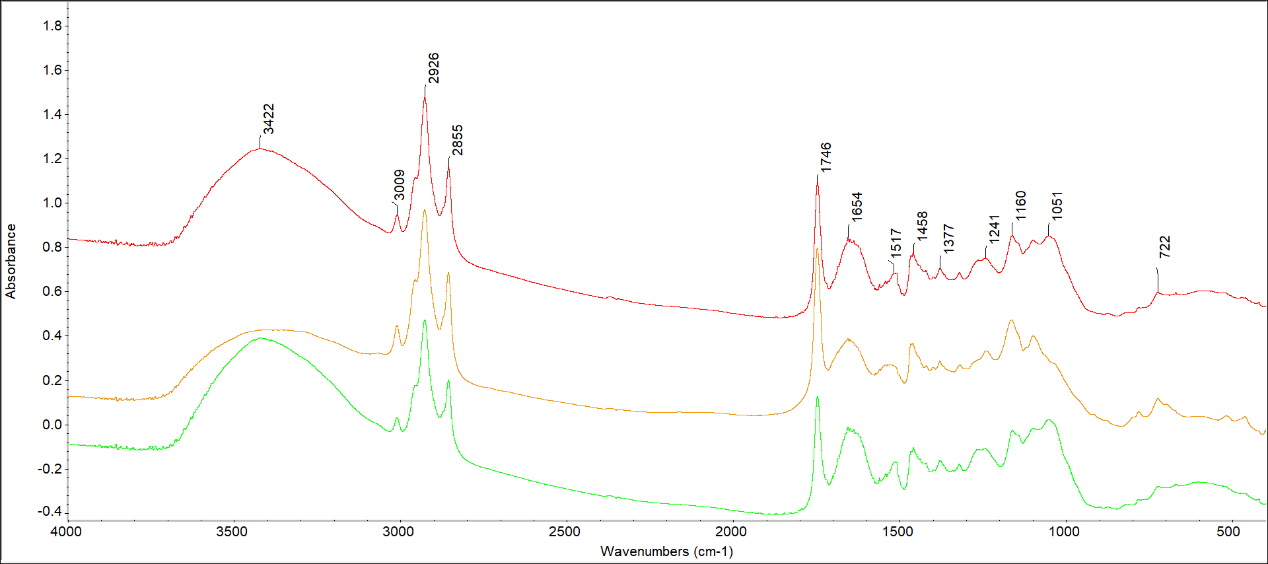


**Figure S1:** FTIR spectra of Cabernet Sauvignon (a) and Tzuriman S. (b) seeds after heating at 150˚C for 2 hours under charring conditions, in comparison to the spectra of fresh Cabernet Sauvignon seed (c).


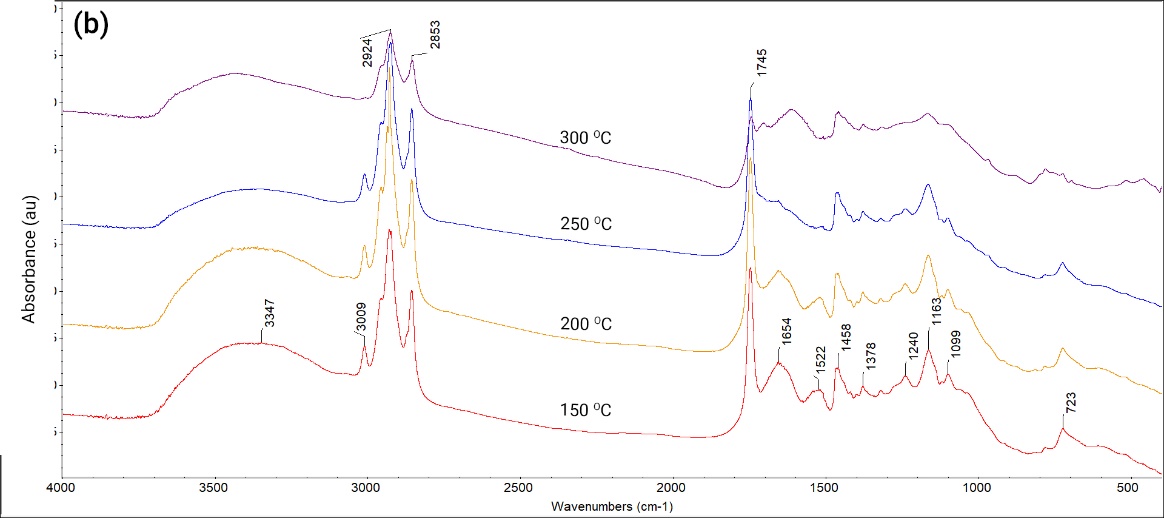

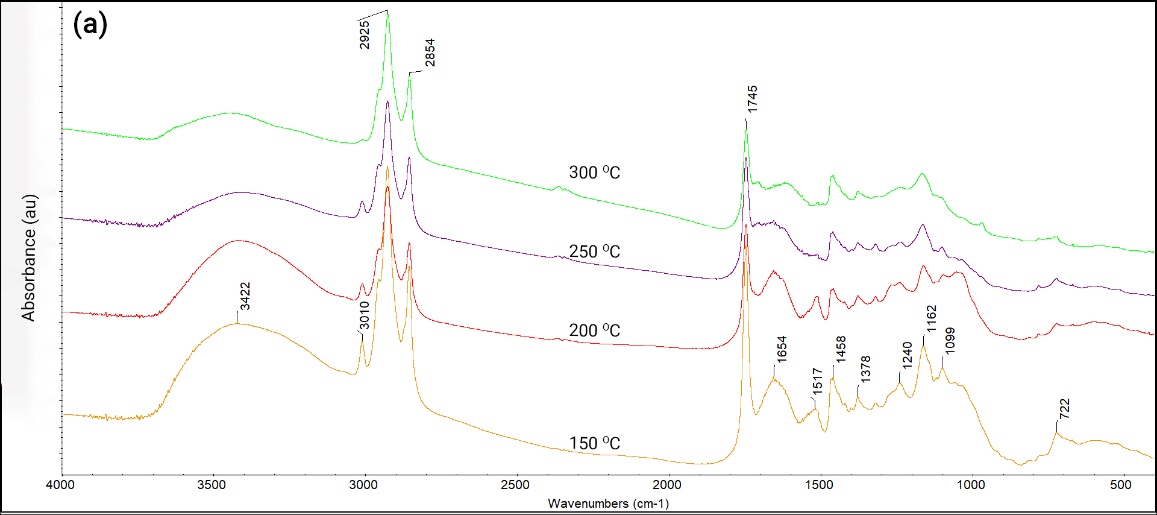


**Figure S2:** A sequence of FTIR spectra of (a) Cabernet Sauvignon and (b) Tzuriman S. 292 seeds after heating at increasing temperatures from 150 to 300˚C under charring conditions.


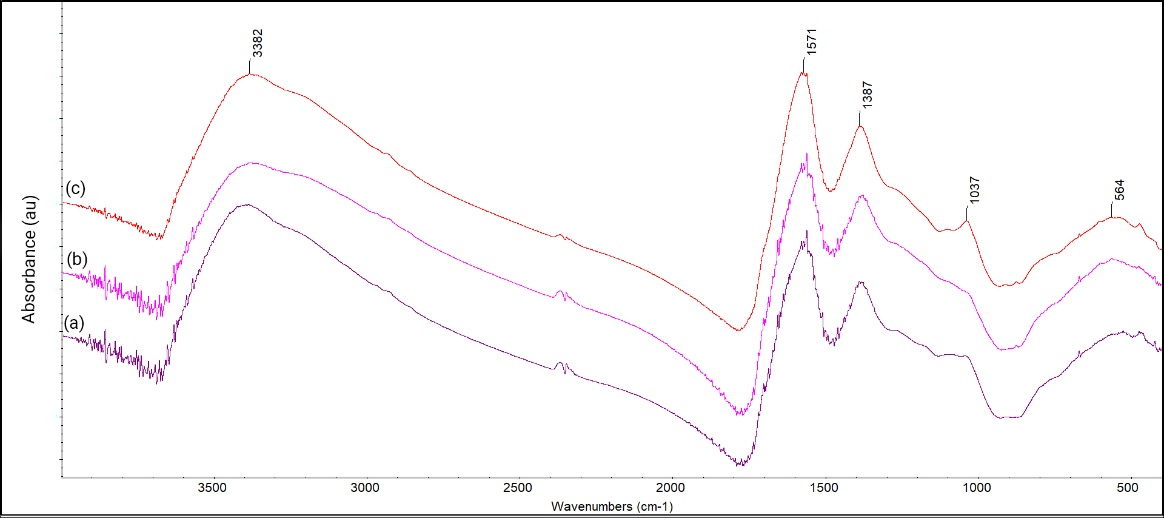


**Figure S3:** FTIR spectrum of archeological grape pip from (a) Beith-El, (b) Ophel and (c) AHD.
