# Supplementary material for: Setting the morphologic quality limits enabling accurate classification of charred archaeological grape seeds
Source: Sci Rep. 2024 Jul 12;14:16148. doi: 10.1038/s41598-024-66896-z (PMC11245623; doi:10.1038/s41598-024-66896-z)
Supplement: Supplementary file 2 — Supplementary Information 2. [file 41598_2024_66896_MOESM2_ESM.docx]

**Not all archaeological specimens are equal - Setting the morphologic quality limits enabling accurate classification of charred archaeological grape seeds.**

Vlad Landa^1^, Yekaterina Shapira^2^, [Adi Eliyahu-Behar](https://scholar.google.com/citations?user=Fy2-jZgAAAAJ&hl=en&oi=ao)^3,4^, Reut Levitan Ben-Arie, Ehud Weiss^5^, Yuval Reuveni^,6,7*^ and Elyashiv Drori^2,8*^

**Appendix B**


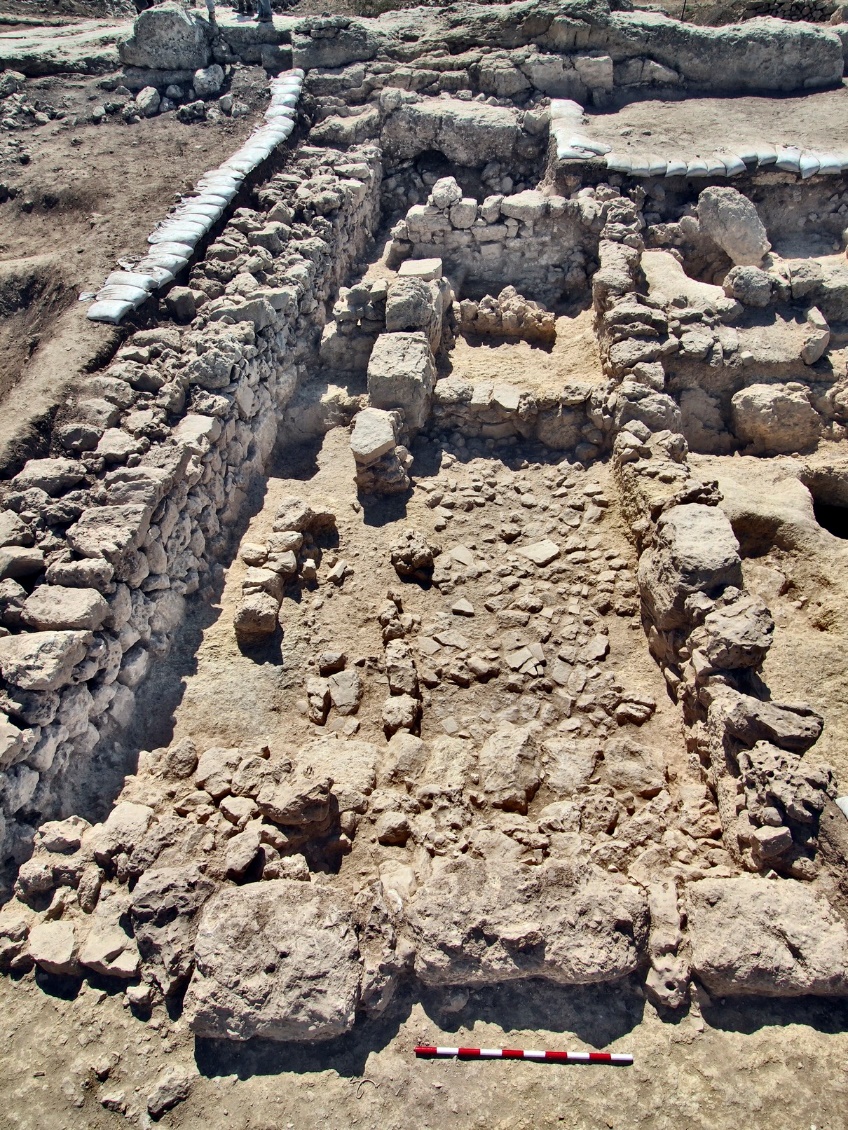


**L8596**

**8578L**

**8568L**

**8560L**

**8595L**

**8561L**

**8580L**

Figure S1- A side photograph of Shilo locus 8561 – the exact point is at the northwestern corner of the building (Photo: Shlomi Amami)


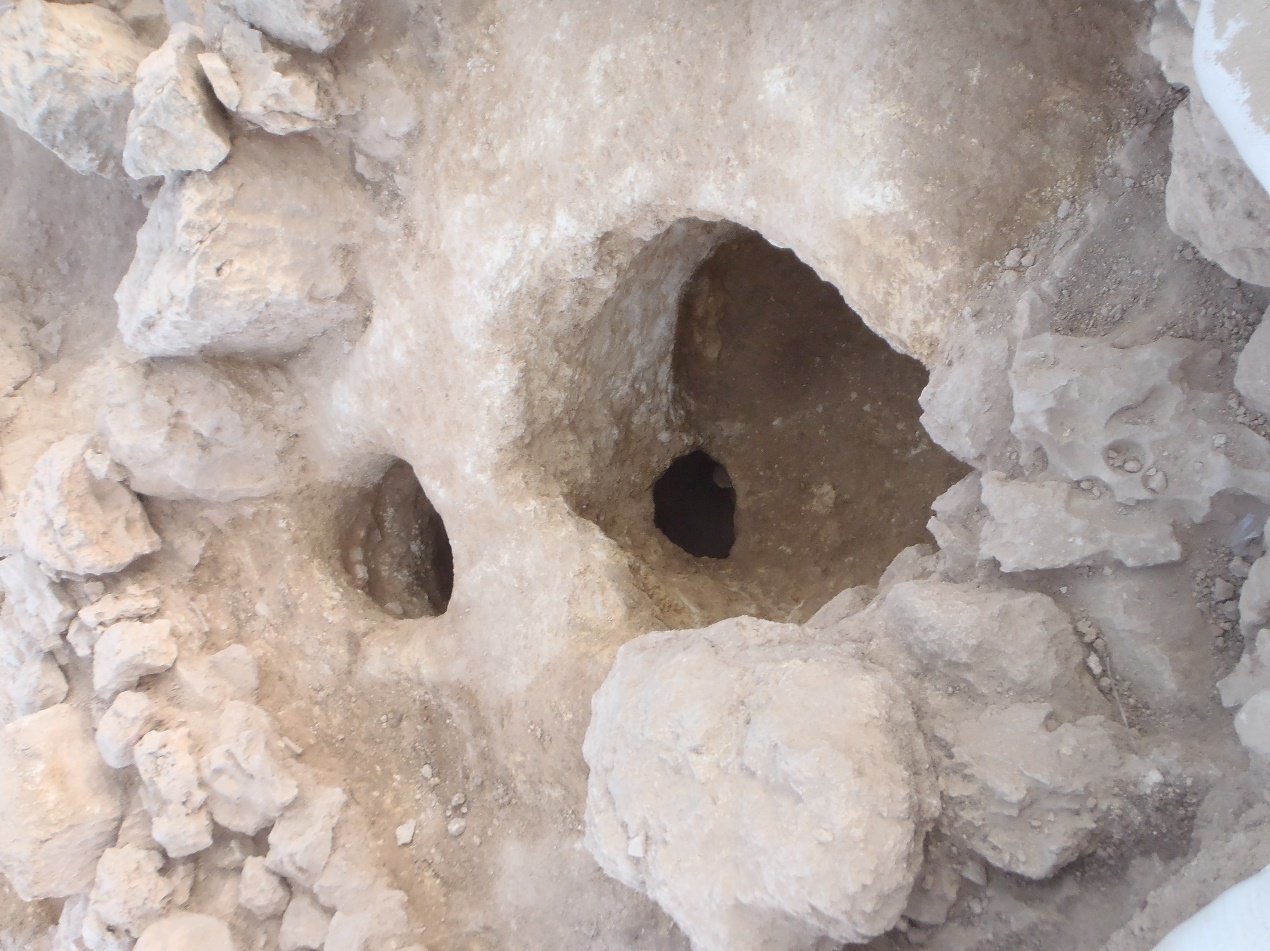


Figure S2- Pits 8583 and 8586 and their connection (Photo: Reut Livyatan Ben Arie).


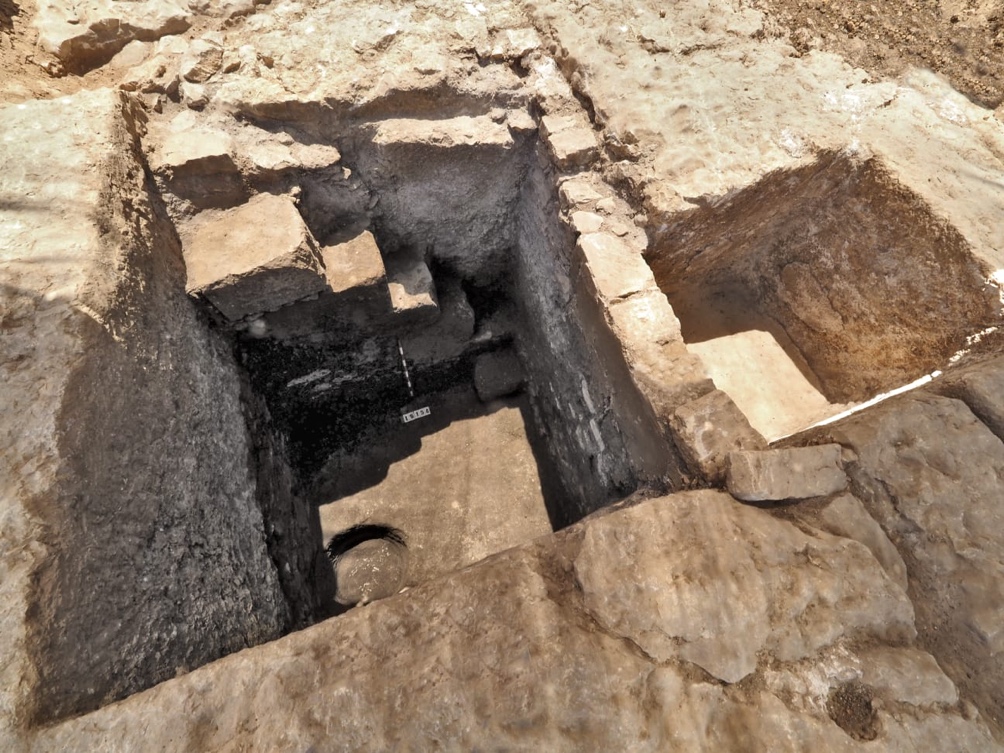


Figure S3. The winepress in Beit El, locus 6154-L
